# Tree-Based Methods for Discovery of Association between Flow Cytometry Data and Clinical Endpoints

**DOI:** 10.1155/2009/235320

**Published:** 2010-01-21

**Authors:** M. Eliot, L. Azzoni, C. Firnhaber, W. Stevens, D. K. Glencross, I. Sanne, L. J. Montaner, A. S. Foulkes

**Affiliations:** ^1^Division of Biostatistics, University of Massachusetts, Amherst, MA 01003, USA; ^2^Immunology Program, Wistar Institute, Philadelphia, PA 19104, USA; ^3^Clinical HIV Research Unit, University of Witwatersrand, Johannesburg, South Africa; ^4^Department of Hematology and Molecular Medicine, National Health Laboratory Service and University of Witwatersrand, Johannesburg, South Africa

## Abstract

We demonstrate the application and comparative interpretations of three tree-based algorithms for the analysis of data arising from flow cytometry: classification and regression trees (CARTs), random forests (RFs), and logic regression (LR). Specifically, we consider the question of what best predicts CD4 T-cell recovery in HIV-1 infected persons starting antiretroviral therapy with CD4 count between 200 and 350 cell/*μ*L. A comparison to a more standard contingency table analysis is provided. While contingency table analysis and RFs provide information on the importance of each potential predictor variable, CART and LR offer additional insight into the combinations of variables that together are predictive of the outcome. In all cases considered, baseline CD3-DR-CD56+CD16+ emerges as an important predictor variable, while the tree-based approaches identify additional variables as potentially informative. Application of tree-based methods to our data suggests that a combination of baseline immune activation states, with emphasis on CD8 T-cell activation, may be a better predictor than any single T-cell/innate cell subset analyzed. Taken together, we show that tree-based methods can be successfully applied to flow cytometry data to better inform and discover associations that may not emerge in the context of a univariate analysis.

## 1. Introduction

Advances in flow cytometry, and particularly technological developments that facilitate acquisition of multiparameter defined phenotypes, present new and exciting opportunities for predicting patient outcomes based on individual specific cell subset changes. This is specifically relevant in the context of studying human immunodeficiency virus (HIV), where there exists a great potential to draw from the rich array of data on host cell-mediated response to infection and drug exposures, to inform and discover patient level determinants of disease progression and/or response to antiretroviral therapy (ART). We describe three existing analytic approaches, designed specifically for uncovering complex structures, and their applications to high density multiparameter cell subset data arising from the use of flow cytometry technology. We demonstrate the usefulness of each approach for novel discovery in this context as well as the contrasting clinical associations that each approach is tailored to address.

The data motivating our research were collected during the pre-randomization stage of the South Africa Structured Treatment Interruption (SASTI) trial, an on-going noninferiority trial that aims to determine whether patients whose ART is interrupted after achieving immune control on therapy will continue to retain the immune reconstitution benefits of therapy. Data on multiple immunological parameters were collected, by way of flow cytometry, on all study participants at start of ART and periodically over the course of the trial. The aim of our present investigation is to illustrate how tree-based machine learning algorithms can be applied to characterize the predictive capacity of a large number of immunological variables, collected at therapy initiation, with regard to a single, clinically relevant measure of immune reconstitution at a fixed time point on continuous therapy and prior to randomization.

We begin by presenting briefly a commonly applied, univariate analysis approach for testing the association between each immunological parameter, individually, and the outcome of interest. We then present three tree-based methods that are designed for discovery of complex structures of association in high-dimensional data settings: (1) classification and regression trees (CARTs) [1]; (2) random forests (RFs) [2]; (3) logic regression (LR) [3, 4]. These methods have been described recently for many high-throughput data settings, including most notably gene chip arrays [5–12]; however, to our knowledge, the application of these analytic approaches to discover predictors of clinical outcomes based on data arising from flow cytometry technologies has not been reported previously.

Notably, the usefulness of CART for immunophenotyping is discussed in Beckman et al. [13], with a review in Boddy et al. [14]. In our setting, the underlying goal differs in that we aim to explore the clinical utility of a large number of a priori defined phenotypes, rather than identify new phenotypes based on a comparatively small number of measurements. Also of note, in an earlier manuscript, Ganju et al. apply CART to identify predictors of censored survival time among patients with cerebral gliomas [15]. Inputs in the analysis include five flow cytometry variables, as well as cytogenetic, molecular and clinical markers. Our investigation extends this research, through consideration of a large number of multiparameter subsets, and by offering a discussion of multiple tree-based approaches, as well as their comparative interpretations, for discovery of associations between these subsets and a clinical endpoint.

## 2. Data and Laboratory Methods

The SASTI trial began in 2006 and led to the successful recruitment of *n* = 127 HIV-1 infected individuals, of whom *n* = 78 individuals completed the 36-week prerandomization phase of the trial. Eligibility criteria for the study included documented HIV-1 infection, 18 years of age or older, and a CD4+ count between 200 and 350 cells/*μ*L in the absence of therapy and within 60 days of the start of the study. All individuals in the trial received a similar ART regimen for the first 36 weeks, and then were randomized to either multiple short-term treatment interruptions or continuous therapy. The present investigation focuses only on prerandomization data, when all subjects are still on ART, as the trial is still on-going as of August 2009.

Cellular immunophenotypes were studied using flow cytometry. Stainings were performed on fresh whole blood at the Department of Hematology and Molecular Medicine, National Health Laboratory Service and University of the Witwatersrand, Johannesburg, South Africa. Briefly, whole blood samples were stained for surface marker detection using fluorochrome-labeled monoclonal antibodies (mAbs) lyophilized on 96-well plates (Lyoplates, BD Biosciences, San Jose, CA). Fluorochrome binding was detected using a 4-color FacsCalibur flow cytometer (BD Biosciences). Cellular subests were analysed using proprietary software (CellQuest, BD Biosciences). Percent of positive cells was calculated based on isotype-matched control mAb binding. Whole blood samples were stained with monoclonal antibody (mAb) combinations (given in Table 1) for 30 minutes, followed by lysis and analysis on a FACScaliber flow cytometer (BD Biosciences). Given the limitation of the instrument (simultaneous detection of 4-color fluorescence), multiple stainings were performed to assess subsets of CD3+ T lymphocytes. The gating strategy is summarized as follows.

(1) Background staining was assessed using isotype-matched mAb (staining 1—this method is generally considered acceptable for surface flow cytometry of lymphocytes).
(2) Postrun electronic event gating was performed using CellQuest software (BD Biosciences), based on the use of multiple 2-color quadrants. A first gating assessed expression of CD3 and CD8 (stainings 2, 3, 4, 6), CD3 and HLA-DR (staining 5), CD3 and CD45 (staining 7), and Lin-1 and HLA-DR (staining 8). Events falling in the quadrants of interest were further gated using quadrants to explore the expression of the remaining markers. The number of events falling in each quadrant was collected. Results are expressed as percent of gated/total events unless otherwise specified.
(3) For T cell subset assessment, the CD4+ T lymphocyte subset was directly stained using CD4 mAb only in staining 7 (single platform CD4 count [16]). Based on the mutually exclusive expression of CD8 and CD4 in the vast majority of T cells (as also assessed in staining 7), in all remaining T cell stainings (2, 3, 4, and 6) CD4+ T cells were defined as CD3+ cells lacking expression of CD8.

In this paper, we focus on assessing the relationships among multiple baseline flow cytometry variables collected at initiation of ART and the variability in achieving a robust CD4+ T-cell count rise on ART, in the context of restricting the range of starting CD4 count between 200 and 350 cells/*μ*L. A complete listing of the baseline flow variables is given in the first column of Table 2. These are fluorescence-based cell phenotypes following intensity threshold gates using two to four fluorochromes. Four replicates, based on independent data acquisitions, were recorded for each of the phenotypes, CD3-CD8-, CD3+CD8-, CD3-CD8+, and CD3+CD8+ and averaged for the analysis. After combining these data, there are a total of 63 flow variables. All variables are measured as a percent of gated at baseline, with the exception of CD4+ which is a cell count. CD4+ T-cells, which are targeted in the viral replication cycle, play an important role in the functioning of the host immune system and are a well-described marker for disease progression when decreasing and as a response to ART based on its inverse relation to viral replication [17]. A CD4+ cell count of greater than 450 cells/*μ*L at 36 weeks on ART is considered a positive response to ART within this study and serves as the outcome in our present investigation. Notably, while this dichotomized version of CD4+ cell count is used in our study, the analytic methods we present are equally applicable to both binary and quantitative outcomes.

## 3. Methods

We present a univariate analysis and three tree-based algorithms. The tree-based approaches involve recursive splitting of the data, based on the value of predictor variables, in a manner that broadly captures the variability in a single outcome. All three approaches are nonparametric and can be applied in the context of a large number of predictors and a single binary or quantitive trait. Both CART and RFs can handle both quantitative and binary predictor variables, while logic regression requires dichotomous inputs. For clarity of presentation, we dichotomize all of the potential predictors a priori. Further discussion of this, including model sensitivity to choice of inputs, is given in Section 5. We begin by briefly defining our notation.

### 3.1. Notation and Univariate Analysis

Suppose we have *p* predictor variables based on the outcome of flow cytometry at a single time point. We denote these with the vector **x**_*i*_ = (*x*_*i*1_,…, *x*_*ip*_) for individual *i*, where *i* = 1,…, *n*. The *n* × *p* matrix **X** is used to denote the full data design matrix with (*i*, *j*)-element corresponding to the value of variable *j* for individual *i*. Subjects are assumed to be independent, though we expect correlation among the predictors. Interest lies in characterizing the association between **X** and a measured trait, which we denote with the vector **y** = (*y*_1_, *y*_2_,…, *y*_*n*_) for the *n* individuals in our study. In our setting, each of the columns of **X**, denoted *X*_*j*_, is a measure of the flow variables and the outcome of interest is a binary indicator for CD4+ cell count >450 cells/*μ*L. We define each *X*_*j*_ as an indicator for being above or below the sample median value for that variable.

Measuring and testing the association between a single categorical predictor and a binary outcome is typically achieved through a contingency table analysis. The odds ratio, defined as the odds of disease given exposure, divided by the odds of disease given no exposure, is a well-described measure of association in the this context and is given formally by

(1) $$\begin{array}{c} OR=\frac{Pr\left(D^{+}\;|\;E^{+}\right)/\left[1-Pr\left(D^{+}\;|\;E^{+}\right)\right]}{Pr\left(D^{+}\;|\;E^{-}\right)/\left[1-Pr\left(D^{+}\;|\;E^{-}\right)\right]} \end{array}.$$

In our setting, we report the odds of having a CD4+ cell count of more than 450 cells/*μ*L (*D* ^+^) given that a specific baseline flow variable is in the upper half of its distribution (*E* ^+^), over the odds of having a CD4+ cell count >450 cells/*μ*L given that this flow variable is in the lower half (*E* ^−^). Pearson's *χ* ^2^-test can be applied as a test of the null hypothesis of no association between exposure and disease for each flow variable independently. An adjustment of the resulting *P*-values, that accounts for the number of tests performed, is needed in this setting for assessing statistical significance. We report the *q*-value which is based on a positive false discovery rate adjustment [18, 19].

### 3.2. Classification and Regression Trees

Classification and Regression Trees (CARTs) are an alternative, nonparametric approach that allows us to model simultaneously the relationship between an outcome and *multiple* potential predictor variables. This approach provides us with information on variable importance as well as the structure of association. Classification trees are constructed for binary outcomes while regression trees apply to continuous traits. Both binary and continuous predictor variables are acceptable inputs, though trees are constructed based on binary splits of these data. The first step in generating a tree is to determine the most predictive variable of the trait, which we denote *X*_(1)_, based on a prespecified splitting rule. Secondly, we divide individuals into groups based on the value of *X*_(1)_ and determine the most predictive variable of the outcome within each of these groups. This process is repeated recursively until a stopping criterion is met and then the resulting tree is pruned back to avoid over-fitting. Tree construction is sensitive to the choice of splitting rule, and ultimately, we want to define such a rule so that we partition our data in a manner that minimizes the within group heterogeneity in the outcome. Here we describe the CART methodology generally, though in the example we present a classification tree since we are considering a binary outcome.

Formally, let the node Ω represent the full set of data and suppose after splitting the data based on one of the predictor variables, we have two groups, Ω_*L*_ and Ω_*R*_, called the left and right daughter nodes, respectively. If the node impurity, or heterogeneity, for Ω is denoted *ℐ*(Ω), then we aim to identify the split that maximizes

(2) $$\begin{array}{c} \phi =ℐ\left(\Omega \right)-ℐ\left(\Omega_{L}\right)-ℐ\left(\Omega_{R}\right). \end{array}$$

That is, we want to choose a split that maximizes the reduction in node impurity. In the context of a binary outcome (*y* = 0 or 1), we let *ℐ*(Ω) = *π*(Ω)*i*(Ω) where *π* is the probability of belonging to Ω, so that (2) reduces to

(3) $$\begin{array}{c} \phi =i\left(\Omega \right)-\pi_{L}i\left(\Omega_{L}\right)-\pi_{R}i\left(\Omega_{R}\right). \end{array}$$

The impurity, *i*(Ω), is commonly measured using the Gini index [12], defined as

(4) $$\begin{array}{c} i\left(\Omega \right)=2p_{\Omega }\left(1-p_{\Omega }\right) \end{array},$$

where *p*_Ω_ = Pr (*y* = 1 ∣ Ω) is the conditional probability that *y* is equal to 1 within the node Ω.

**Figure 1:**
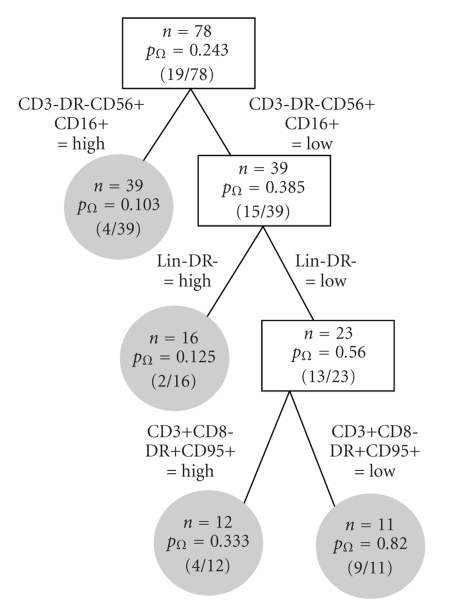
Classification tree (unpruned).

Once a tree is constructed, as shown in Figure 1, we prune it to ensure its applicability to external datasets. Importantly, increasing the number of splits in a tree will inevitably decrease the prediction error for the data used to generate the tree. However, a smaller tree may better describe the underlying structure in the population at large. Therefore, after we build a tree, as described above, we prune it in order to get an optimal subtree, using cost-complexity pruning. Briefly, for tree *𝒯* of size |*𝒯*| and complexity parameter *α* ≥ 0, the cost complexity is given by

(5) $$\begin{array}{c} R_{\alpha }=R\left(𝒯\right)+\alpha \left|𝒯\right|, \end{array}$$

where

(6) $$\begin{array}{c} R\left(𝒯\right)=\underset{\tau \in \tilde{𝒯}}{\sum }\text{Pr}\;\left(\tau \right)r\left(\tau \right), \end{array}$$

$\tilde{𝒯}$ is the set of terminal nodes in tree *𝒯* and *r*(*τ*) is the measure of error for the node *τ*. In the case of a binary outcome, we let *r*(*τ*) equal the misclassification rate.

### 3.3. Random Forests

Random Forest (RF), originally proposed by [2], is an alternative approach that involves generating a collection of trees. Since this approach results in an ensemble of trees, which tend to vary in structure, RFs serve to quantify the importance of variables, rather than depicting the specific structure of association among variables. A primary advantage of RFs is that, through sampling a subset of variables at each split, it offers a natural approach to handling collinearity among the predictors. In this paper, we demonstrate the application of RFs as an exploratory tool, although methods for determining statistical significance based on variable importance scores have been described recently [20, 21].

The RF algorithm is summarized by the following step-by-step procedure: (1) generate a learning sample by sampling *n*_1_ individuals with replacement from our data (usually about two-thirds of the data). We call the remaining *n*_2_ ≈ *n* − *n*_1_ data the out-of-bag (OOB) data; (2) using the learning sample data, generate an unpruned tree by randomly sampling a subset of the predictors at that node. These predictors will be used as our variables on which our splitting decisions are based (3) based on the OOB data, find the overall tree impurity, and call this *π*_*b*_. Permute the predictor *X*_*j*_ and record the overall tree impurity for each *j* = 1,…, *p*. Call tree impurity for the *j*th predictor *π*_*bj*_ and call variable importance for this predictor *δ*_*bj*_ = *π*_*bj*_ − *π*_*b*_. (4) repeat steps (1)–(3) for *b* = 2,…, *B* in order to obtain *δ*_1*j*_,…, *δ*_*Bj*_ for each *j*.

For each predictor, *j*, the overall variable importance score is given by the average importance over the *B* trees. Formally, we write

(7) $$\begin{array}{c} {\hat{\theta }}_{j}=\frac{1}{B}\overset{B}{\underset{b=1}{\sum }}\delta_{bj}. \end{array}$$

Notably, for each tree, a learning sample is used in the tree construction, while an independent test sample, called the OOB data, is used to evaluation variable importance.

### 3.4. Logic Regression

Logic regression (LR) is another tree-based approach that is increasingly popular for the analysis of high-dimensional data. LR searches specifically for models that are comprised of combinations of Boolean expressions of the predictors [3, 4]. Boolean expressions take on the value of either 0 or 1, and are themselves functions of binary variables, related to each other by “and,” “or,” and “complement” statements. Formally, LR models are of the form

(8) $$\begin{array}{c} g\left(E\left[Y\;|\;X\right]\right)=\beta_{0}+\overset{t}{\underset{j=1}{\sum }}\beta_{j}L_{j} \end{array},$$

where *L*_*j*_ is a Boolean combination of the binary predictors. Suppose that we have binary predictor variables *X*_1_, *X*_2_,…, *X*_*p*_ which we want to use to predict some outcome. An example of a Boolean expression in terms of our group of predictors is (*X*_1_⋀*X*_2_)∨(*X*_3_⋀*X*_4_^*c*^), which represents “both *X*_1_ = 1 and *X*_2_ = 1 or both *X*_3_ = 1 and *X*_4_ = 0.”

## 4. Example

We report the results of applying a univariate analysis and each of the tree-based methods described above to data arising from the SASTI trial detailed in Section 2. In total, *n* = 63 flow cytometry variables, measured at baseline, are used as potential predictors (in addition to CD4+ count at baseline). Each variable is dichotomized to indicate whether the value is above or below the median of the observed (nonmissing) values for that predictor. That is, an observation is set equal to 1 if it is greater than the median value for all observations in our sample of that predictor and 0 otherwise. A single imputation is used such that missing data points are assigned the most common value of 0 or 1, based on the nonmissing data for the corresponding variable. The outcome of our analysis is an indicator for whether CD4+ cell count is greater then 450 cells/*μ*L at 36 weeks after initiation of ART, which represents the last time point prior to randomization.

The univariate analysis results are provided in Table 2. Here the OR is reported as a measure of association between each flow variable at baseline and CD4+ cell count at 36 weeks on ART. The *P*-value corresponds to Pearson's *χ* ^2^-test of association. Based on this analysis, we see that CD3-DR-CD56+CD16+ is the most predictive variable with an OR = 0.183 (unadjusted *P* = .008). This suggests that the odds of having a CD4+ cell count >450 cells/*μ*L while on therapy is higher among individuals with a baseline observed CD3-DR-CD56+CD16+ that is in the lower half of our sample. Lin-DR- at baseline is the next most predictive variable, with an OR = 0.23 (unadjusted *P* = .018). After adjusting for multiple testing using the approach of Benjamini and Yekutieli [22], we cannot conclude that any of the flow variables alone are significantly associated with CD4+ count after 36 weeks. The repeated ORs reported in this table are likely due to the limited sample size in our study, as clear relationships among these pairs and triplets of variables are not generally well-established.

An unpruned classification tree, based on a stopping rule of *n* = 5 individuals per node, is illustrated in Figure 1. This model yields five terminal nodes, indicated by the shaded circles, resulting from splits based on CD3-DR-CD56+CD16+, Lin-DR- and CD3+CD8-DR+CD95+. The first split indicates, for example, that for high CD3-DR-CD56+CD16+ (i.e., CD3-DR-CD56+CD16+ greater than the median), only *p*_Ω_ = 4/39 = 10.3% of the individuals in our sample have an observed CD4+ count that is greater than 450, while for low CD3-DR-CD56+CD16+ (i.e., CD3-DR-CD56+CD16+ less than the median), *p*_Ω_ = 15/39 = 38.5% of individuals have a CD4+ cell count that is greater than 450 cells/*μ*L. Among those individuals who fall to the right daughter node (i.e., low CD3-DR-CD56+CD16+), the next most important predictor is Lin-DR-. When CD3-DR-CD56+CD16+ is low and Lin-DR- is high, 2/16 = 12.5% of the subjects in our sample have an observed CD4+ count that is greater than 450. On the other hand, when both CD3-DR-CD56+CD16+ and Lin-DR- are low, a much higher percentage (13/23 = 56.5%) of individuals have a CD4+ count greater than 450 cells/*μ*L. Application of cost-complexity pruning resulted in a tree with no splits, suggesting that these findings may not be reproducible in an independent sample. This may be a consequence of limited power in our small sample setting.

**Figure 2:**
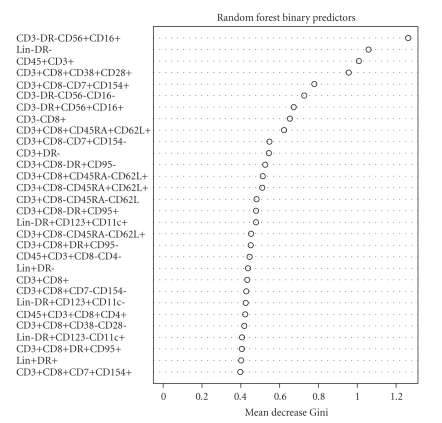
Variable importance scores from application of an RF.

The results of applying the RF algorithm to these data are given in Figure 2. Here we see that the most important baseline predictor of CD4+ count on ART is again CD3-DR-CD56+CD16+, with a mean decrease in node impurity of 1.26. The next most important variable is Lin-DR- (also the second split in our classification tree), with a corresponding mean decrease in node impurity of 1.05. These results are generally consistent with the univariate analysis of Table 2 and to some extent with the classification tree of Figure 1; however, some notable differences are apparent. First, the RF analysis places more emphasis on CD45+CD3+ as an important predictor than the CART analysis. Interestingly, CD45+CD3+ is also the third most important variable in the univariate analysis. Since the classification tree is considering a series of conditional analyses, this difference may be a result of CD45+CD3+ not having a strong association *within* levels of the first splitting variable, CD3-DR-CD56+CD16+. Secondly, the classification tree analysis places greater emphasis on CD3+CD8-DR+CD95+ than either the RF or univariate approaches. This specifically lends some insight into a potential effect of the combination of CD3-DR-CD56+CD16+, Lin-DR-, and CD3+CD8-DR+CD95+.

**Figure 3:**
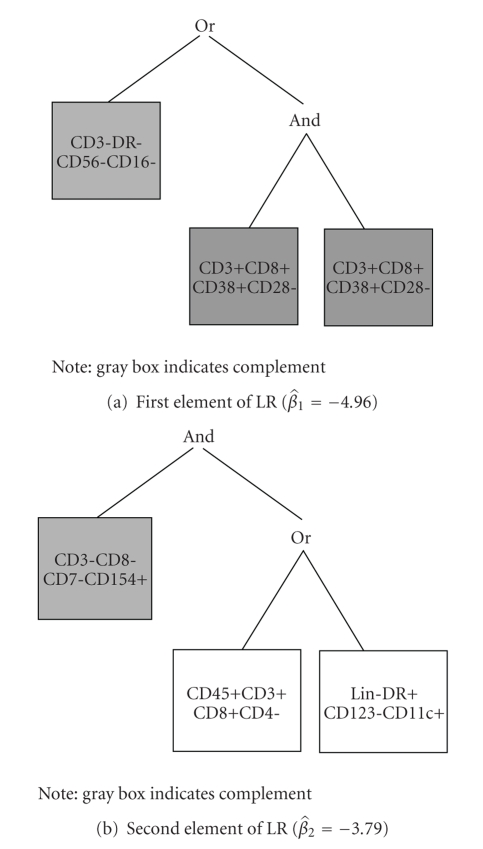
Logic regression trees.

Finally, we applied LR to the data and the resulting trees are presented in Figure 3. Here we applied a logit link function, specified that we wanted two trees and restricted the total number of “leaves” (across both trees) to 6 for ease if interpretation. The coefficient estimates for the trees in Figures 3(a) and 3(b) are ${\hat{\beta }}_{1}=-4.96$ and ${\hat{\beta }}_{2}=-3.79$, respectively. In this case, the variable CD3-DR-CD56-CD16- is an important predictor of CD4+ count on therapy. Notably, this variable is highly negatively correlated with CD3-DR-CD56+CD16+ (Pearson's *ρ* = −0.71), which was identified as the most important predictor of immune reconstitution based on the other approaches described above. In addition to CD3-DR-CD56-CD16- being an important predictor of immune reconstitution, we have, for example, based on the second tree, that when CD3+CD8-CD7+CD154+ is low (less than the median) and either CD45+CD3+CD8+CD4- or Lin-DR+CD123-CD11c+ is high (greater than the median) the log odds that CD4+ count is greater than 450 cells/*μ*L decreases by 3.79, compared to when this does not hold.

## 5. Discussion

The goal of this study is to compare a number of tree-based methods for their capability to select immunological predictors of CD4 reconstitution in HIV-infected subjects initiating antiretroviral treatment. Earlier studies from our group have demonstrated that pre-ART CD95 expression on CD8+ T cells is negatively associated with the frequency of plasmacytoid Dendritic Cells (PDCs) after 52 weeks of treatment [23]. Conversely, a positive association was also demonstrated between levels of baseline CD28 expression in CD4+ T cells and PDC recovery. Other studies have also suggested that baseline CD4 count may predict the degree of post-ART immune reconstitution [24]. However, the selection of immunologic predictors of immune reconstitution has so far been based on known biologic associations between variables (e.g., association of a certain variable with diseases stages, etc.), and data-mining methods for automated unbiased selection from a large numbers of variables remain underutilized.

We describe the application of a univariate approach and three tree-based methods for the analysis of the association between a single trait and multiple variables arising from flow cytometric analysis. Interestingly, for this data example, the univariate contingency table analysis and RFs resulted in similar findings in terms of the ranking of important variables. This may not always be the case, since as we describe in Section 3, the variable importance scores derived within the context of RFs are based on the individual effects of variables, as well as their effects within levels of other variables. In the example provided, CART and LR provided complementary information about the structure of association, and particularly the combinations of variables that are informative. Specifically, while all of the approaches suggest that CD3-DR-CD56+CD16+ is an important predictor of CD4+ count on therapy, the CART model further suggests that among individuals for whom CD3-DR-CD56+CD16+ is in the lower half of our sample, Lin-DR- is an important variable in differentiating between responders and nonresponders. Similarly, the LR analysis revealed several combinations of variables that lend further insight into determining the individual level characteristics that together are predictive of response to ART in this population. The added information on variables that are predictive of outcome, beyond those identified by univariate analysis, provides greater understanding of multiple combinations among variables that may equally predict an outcome, reflecting the potential complexity of responses among human study groups.

Notably, a high degree of correlation is intrinsic to the variables included in our analysis of flow cytometry data. Specifically, events passing a certain logical gate are assessed for co-expression of two fluorochromes, and separated in quadrants based on the intensity (above or below a certain level) of each fluorochrome. Thus, any increase in the percent of events falling in one quadrant must correspond to a decrease in the percent of events that fall in one or more of the other quadrants. For example, the variables CD3-CD8-, CD3+CD8-, CD3-CD8+, and CD3+CD8+ arise from four quadrants on the same plate for each individual and thus always sum to 100%. While each variable represents a distinct cell subset, and application of the described approaches to data with such a correlation structure is reasonable, further extensions of these methods that account for the correlation structure may offer new insights. At the same time, interpreting variable importance must be done in light of the existing correlations. For example, we saw in the example provided above that both CART and the RF identified CD3-DR-CD56+CD16+ as an important predictor of immune reconstitution, while LR identified the highly correlated variable, CD3-DR-CD56-CD16-. RFs offer a natural approach to handling correlations by sampling a subset of predictors at each stage of the tree splitting; however, using any of the approaches described, the importance of a variable may be obscured in the presence of other, very highly correlated variables. One alternative approach is to choose a priori a subset of uncorrelated variables to include in the analysis. This is reasonable if prior knowledge suggests multiple variables are defining the same underlying construct but may be less optimal if the precise relationship among variables is unknown.

This paper represents an attempt to utilize data from experimental and clinical laboratory settings that are available in resource constrained settings. While it is general good scientific practice to avoid unnecessary assessment, limiting stainings and maximizing the usefulness of current resource capacity is paramount in the settings in which these experiments were conducted. Because the use of multicolor flow cytometers is restricted to resource-rich clinical and research settings, we have elected to use the output of more commonly available 4-colour analytical instruments, in the hope that any information gained from this approach is applicable in the resource-constrained settings such as those in which the study was conducted. We also agree that the clinical interpretability of the findings in this data setting is limited. Specifically, the full panel of mAb used for this paper would not be applicable to general practice, particularly in resource constrained settings, due to issues of cost and laboratory capacity. This panel was in fact used in an experimental setting, to investigate in detail the effects of ART on individual immune subsets. However, the purpose of this paper is to identify which, among the baseline, pre-ART stainings performed, could be useful to predict the desired outcome (in this case immune reconstitution as assessed by CD4 counts). We demonstrated how tree-based approaches can be applied to identify a small number of phenotypes that contribute to the selected CD4 recovery outcome. Importantly, many of the cellular subsets (e.g., mature NK cells, myeloid Dendritic cells, CD95-expressiong activated T lymphocytes) selected using the three tree-based methods presented here as being predictive of immune reconstitution have been previously shown to be individually correlated with disease progression and/or immune reconstitution [25, 26], thus further supporting the reasonableness of our approach. CD4 is presently the only validated tool to monitor immune competence in HIV-infected individuals. However, because pre-ART CD4 counts are notoriously poor predictors of clinical response to ART, the identification of a limited number of variables that could be used as additional predictors in larger prospective studies represents an important contribution to the field. Selected stainings can be recombined in smaller panels, reducing cost and capacity consumption; for example, based on the logic regression trees presented in Figure 3, the use of only two staining combinations (e.g., CD3/CD8/CD7/CD154 and CD45/CD3/CD4/CD8) would be sufficient to predict a CD4 immune reconstitution outcome.

Importantly, differences in the insights offered by each of the approaches presented are a reflection of the specific algorithms employed and not the result of one approach being more or less correct than another. The univariate analysis, while methodologically sound, only considers associations that exist between single variables and the outcome. Univariate analyses are not designed to discover variables that are only important conditional on the level of another variable. The CART and RF algorithms, on the other hand, are specifically searching for conditional associations, that is, associations of variables with the outcome within levels of other variables. Finally, logic regression trees allow for discovery of combinations of variables that are predictive, even in the setting in which no single element of the combination is important on its own. That is, both CART and RF split initially on the single most important variable; however, if a combination of two or more variables is important, none of which are predictive individually, then both CART and RF may not find this association [12, 27]. The LR algorithm, on the other hand, is designed specifically to capture this information.

In summary, each of the tree-based approaches described herein complement univariate analyses of multiparameter defined flow cytometry subsets. These methods are designed specifically to uncover complex structures, and as demonstrated in the example above, allow for discovery of combinations of variables that are together predictive of an outcome. While extensions of these methods, including, for example, the recently proposed approach of [20], would allow for measuring statistical significance of variable importance scores, their strength lies in the discovery of combinations of variables that are potentially associated with the outcome. In all of the approaches presented, a type of cross-validation algorithm is applied, which renders the results theoretically applicable to independent samples. However, as with all exploratory analyses, further hypothesis driven research will enable further validation of true underlying associations.

## Figures and Tables

**Table 1:** 4 Color stainings employed for flow cytometric analysis.

Staining no.	FITC	PE	PerCP cy5.5	APC
1	Ig	Ig	Ig	Ig
2	CD45RA	CD62L	CD3	CD8
3	CD38	CD28	CD3	CD8
4	HLA-DR	CD95	CD3	CD8
5	CD56	CD16	CD3	HLA-DR
6	CD7	CD154	CD3	CD8
7	CD8	CD4	CD45	CD3
8	Lin-1	CD123	HLA-DR	CD11c

**Table 2:** Univariate associations with CD4+ count at 36 weeks on ART.

Predictor	Odds ratio	*P*-value
CD3-DR-CD56+CD16+	0.183	.008
Lin-DR-	0.228	.018
CD45+CD3+	0.274	.035
CD3+CD8+CD38+CD28+	0.281	.047
CD3-CD8+	0.323	.084
CD3-DR+CD56+CD16+	0.339	.087
CD3+CD8-CD7+CD154+	0.339	.087
CD3-DR-CD56-CD16-	0.364	.113
CD3+CD8+CD7+CD154+	0.388	.463
CD3+CD8-CD7+CD154-	0.389	.146
CD3+CD8-DR+CD95-	0.429	.189
CD3+DR-	0.460	.236
CD3+CD8-CD45RA+CD62L+	0.477	.283
CD3+CD8-DR+CD95+	0.477	.283
CD45-CD3+	0.494	.632
CD3+CD8-	0.494	.632
Lin-DR+CD123+CD11c+	0.564	.424
CD3+CD8+DR+CD95+	0.628	.571
CD3-DR+	0.646	.586
CD3+CD8-DR-CD95-	0.646	.586
CD45+CD3+CD8-CD4-	0.703	.690
CD3+CD8+CD38-CD28+	0.709	.699
CD3+CD8-CD7-CD154-	0.740	.774
CD3+CD8+	0.752	.786
CD3+CD8-CD38+CD28+	0.759	.797
CD3+CD8+CD38+CD28-	0.760	.812
CD3-DR+CD56-CD16-	0.805	.887
CD3-DR+CD56+CD16-	0.805	.887
CD3+CD8+CD38-CD28-	0.813	.898
CD45-CD3-	0.862	.989
Lin-DR+CD123+CD11c-	0.913	.917
CD3+CD8+CD45RA+CD62L-	0.923	.908
CD3+CD8+CD45RA-CD62L	0.931	.898
CD45+CD3+CD8-CD4+	0.931	.898
CD3+CD8+DR+CD95-	0.938	.887
CD3+CD8-CD7-CD154+	0.962	.696
CD3+CD8-CD38+CD28-	0.996	.797
CD3+CD8+DR-CD95-	1.004	.797
Lin+DR+	1.074	.898
CD3-DR-CD56-CD16+	1.074	.898
CD3-CD8-	1.074	.898
Lin+DR-	1.149	1.000
CD45+CD3-	1.160	.989
CD3+CD8-CD38-CD28-	1.160	.989
CD3+CD8-CD45RA+CD62L-	1.230	.898
CD45+CD3+CD8+CD4-	1.317	.797
CD3+DR+	1.329	.786
Lin-DR+CD123-CD11c-	1.329	.786
CD3+CD8-DR-CD95+	1.329	.786
CD3+CD8+DR-CD95+	1.410	.699
CD45+CD3+CD8+CD4+	1.410	.699
CD3+CD8+CD45RA-CD62L+	1.422	.690
CD3-DR-	1.446	.677
CD3-DR+CD56-CD16+	1.486	.661
Lin-DR+	1.511	.605
CD4+	1.522	.598
Lin-DR+CD123-CD11c+	1.522	.598
CD3+CD8-CD45RA-CD62L+	1.630	.512
CD3-DR-CD56+CD16-	1.630	.512
CD3+CD8+CD7+CD154-	1.657	.502
CD3+CD8+CD7-CD154-	1.707	.487
CD3+CD8-CD38-CD28+	1.898	.354
CD3+CD8-CD45RA-CD62L	2.011	.294
CD3+CD8+CD45RA+CD62L+	2.152	.238
